# A Case of Enlargement of the Thyroid Gland, Treated by Seton

**Published:** 1847-07

**Authors:** Henry Kennedy


*A case of Enlargement of the Thyroid Gland, treated by Seton.* By Henry Kennedy, M. D.—In November, 1845, a woman, aged 35, applied to me on account of an enlarged thyroid gland. She had been married nine years, and had four children; she has lived of late years in Dublin, and has always been healthy in every respect, excepting the disease she applied about. The gland had begun to enlarge so far back as the year 1832, thirteen years before my seeing her. At first it had increased very slowly; but the last year or so, she said, it grew more rapidly. When I saw it the tumour was at least the size of the largest orange; it was very hard to the touch, as if it were solid, but was divided into two portions, of which that on the right side was much the largest; it did not vary in size at the menstrual periods. It was not, however, on account of the bulk of the tumour, for in that respect there was nothing remarkable, that the patient applied for relief, but because it had affected her swallowing from a very early stage of its growth; and this symptom had latterly become much more distressing: solids were more difficult to get down than fluids, as might be expected. She referred the obstruction to the seat of the tumour. She told me she had shown it to other medical men, but she considered it still increasing. I ascertained that iodine had lately been used, both internally and externally for some weeks.

Under all the circumstances of the case, the tumour and dysphagia on the increase, and iodine having got a full trial, I determined on some more decided line of treatment, the more readily as the patient herself was most anxious that something should be done. The plan by seton seemed to hold out the best prospect of success, and it was carried into effect, having previously brought the health to the best condition. The first seton was passed on the 30th of November, 1845. A common curved needle of the largest size, with its eye nearly full of double silk thread, was passed from below directly upwards, through the anterior portion of the tumour, about half an inch from the middle line of the neck, and including a space of at least one inch and a quarter between the entrance and exit of the seton. This was then fixed so as to prevent its slipping out, and the patient was desired to keep a poultice constantly applied, and also to keep her bed for two days; no unpleasant effects followed. It is enough to state here that this first seton was withdrawn at the end of ten days; that at the end of a fortnight a second one was passed; that it was double the size of the first, and its introduction was followed by a very considerable degree of constitutional irritation, which, however, subsided in about four days; suppuration then became very fully established, and the seton was withdraw, after being in twelve days. With the exception of poulticing, nothing was done during the next four months. In this time considerable changes had taken place in all the anterior portion of the tumour, and that part of it which occupied the left side; it had become very hard, and gradually, but steadily diminished in size. The larger portion of the tumour, however, occupying the right side of the neck, remained stationary. It appeared, indeed, as if it had grown somewhat larger; but this was not certain. A third and last seton was passed through this portion of the tumour in the month of April, 1846; its direction was upwards and outwards, so as to take in the longer axis of the swelling. This seton was four times larger than the previous one; it was passed with a large packing-needle, with the edges and point properly ground. After sixteen days the seton was withdrawn, the suppuration being then very considerable. Finally, after four months more, the entire tumour had so much lessened, that it might be considered as cured. The entire process occupied between eight and nine months.

At the present time (January, 1847) the eye cannot detect any tumour, but to the touch one remains, which is probably the size of a small chestnut. There is no deformity whatever, and very trifling marks of where the setons had been passed. The patient, too, feels no difficulty of swallowing, at least none that causes any inconvenience.

As I wish here to confine myself merely to the facts of this case, I have purposely omitted the consideration of several points which might fairly admit of discussion; such as the nature of the tumour; the question of a more general use of this plan, after the more ordinary means have failed, particularly iodine; the nature of the dysphagia, as to whether it was nervous or mechanical; the causes of those enlargements, and other points connected with the subject in a general way. It is to Quadri, of Naples, that we are indebted for the plan of treatment put in force in the present case. Not being certain of what the result of the treatment would here be, I did not take the precaution of getting a cast of the tumour when it was of large size. This, probably, is of less consequence, as the patient has been seen by several gentlemen to whom I may here refer. Dr. Clarke, of Herbert street, saw her repeatedly; he took much interest in the case, and kindly gave me his assistance. At a late stage, and after all the setons were withdrawn, the patient was seen by several physicians and surgeons of this city.—*Dub. Quar. Journ.*

